# Buffalo Academy of Medicine

**Published:** 1896-06

**Authors:** Lillian Craig Randall

**Affiliations:** Secretary


### Society Proceedings.

### BUFFALO ACADEMY OF MEDICINE.

### GYNECOLOGICAL AND OBSTETRICAL SECTION.

Reported by LILLIAN CRAIG RANDALL, M. D , Secretary.

THE regular monthly meeting of the obstetrical and gynecological section of the Academy of Medicine was held at the Academy parlors, Tuesday, April 28, 1896.

In absence of the president, Dr. Frederick, Dr. J. W. Grosvenor was called to the chair. Thirty-five members were present during the evening.

The minutes of the previous meeting were read and approved.

### THE TEACHING OF OBSTETRICS.

Dr. M. A. Crockett read a paper with the above title. In this paper the lecture and recitation methods of teaching medicine are compared, the subject of obstetrics being taken for illustration.

The properly conducted recitation is that in which both interest and thoughtfulness are aroused by making the student play an active part. The plan of giving out certain pages in a book and asking questions merely to test the retentive power is condemned as unworthy the name of teaching. Yet such a method has been introduced into medical schools. The scientific recitation consists of four stages: clearness, association, generalisation and application. The first step is a brief review of the subject-matter which forms the elements of the recitation. In the second stage the class puts the facts into various relations. In the writer's class during the past year the woman in labor was taken as the starting point; the physiology of labor was learned, and from the facts, placenta previa, rupture of the uterus, accidental hemorrhage and other forms of dystocia were discussed by the class without the use of the text-book. In the same manner the third stage is carried out, and the class thinks out principles from the facts which are given. The fourth stage deals with the application of these principles. Association and generalisation must be done by the students themselves. Telling is not teaching, and the student can become thoughtful only by being made to think. The writer does not maintain that the students can escape the memorising of certain facts, but as it is now they are not taught how to economise effort. The lecture system deals primarily with subject-matter and treats the mind as something to be filled up. In the recitation the student does his mental work every day in the class-room, but under the lecture system he waits until the end of the year and then crams. The writer pointed out how poor teaching is almost universally the result of poor method and not lack of knowledge on the part of the instructor; yet recitations are given in charge of the youngest and most inexperienced men. Lecturing is extremely easy and demands but little ability compared with that demanded of an instructor. To plan a recitation, ask skilful questions, direct the thought of the class and awake discussion and mental activity constitute some of the requirements of a good instructor. The lecture was proper in medieval times, when a man could tell his hearers what they could find in no book, and is retained at the present day because men like to pose before students and hear themselves talk. If we must have lectures for the benefit of the lecturers, the writer begs for a greater number of scientifically conducted recitations for the benefit of the students.

In conclusion the writer outlined the course in obstetrics proper for a four years' curriculum. He advises the developmental plan of recitation as outlined above, combined with clinical and operative exercise.

The principles of teaching rest upon the manner in which the mind acquires the best kind of knowledge. It will be a happy day for students when the medical faculties begin to regard those principles rather than individual aggrandisement.

Dr. P. W. Van Peyma, in opening the discussion, agreed in a general way with Dr. Crockett's most scholarly paper, a subject logically and truthfully presented. He himself would rather be a teacher than a lecturer. Times have passed when these things had to be given in dictation in didactic lectures. Too much material is crowded into limited time. More time should be given obstetrics. In Leopold's clinic, in Dresden, the class was divided into groups. After fifteen minutes' study of case, students were asked for their diagnosis. A certain amount of knowledge must be assimilated under careful clinical instruction.

Dr. Eugene A. Smith agreed with the essayist especially in regard to the fact that lecturers and teachers so-called loaded up themselves with certain facts to discharge upon student, but he did think that a certain amount of didactic teaching was necessary. He could not see how anatomy could be presented without the students doing a certain amount of work. In obstetrics the real instructor could spend an hour in developing a certain definite obstetrical truth, but unless the student has a knowledge of anatomy gleaned from personal study, he cannot use these truths.

Dr. Frederick said his teaching experience had been clinical and believed that theoretical obstetrical teaching should be eliminated and teaching of obstetrics should be clinical, not didactic. Teaching must be modified by each man's personality and experience. His own teaching was from his own experience and could not be found in any book.

Dr. J. W. Grosvenor: The American Academy of Medicine has occupied itself with this question and has induced many teachers to take up the recitation method.

Dr. J. H. Pryor said he enjoyed the paper for its thoughtfulness and literary merit. Teaching has been rank in our medical colleges for the reason that the medical men who are at their head conduct them, both hospitals and colleges, as business concerns, and these medical men like to hear themselves talk. Often professors will give their own treatment, which is of no value. Often in the lecture so much is crowded into an hour that the student cannot grasp single facts.

Clear-headed young men who are examined for the Erie County Hospital show how little they have absorbed and how they would learn had they a chance. Something is radically wrong with the teaching. He hoped the day would come when the didactic lecture will be done away with. This should be done in the interest of the student, not the professor.

Dr. Crockett, in closing the discussion, said he was much obliged to the speakers for this hearty discussion. As Dr. Pryor has said, it is not the trend of thought of the professor, it is the trend of thought of the student.

He does not entirely condemn all didactic lectures or instruction. Anatomy, a hard study to teach, should be taught from the patient and in the dissecting-room. Ideal recitation is an end toward which teachers must aim.

### SEPTICEMIA IN THE NEW-BORN.

Dr. Snow read a paper with this title. This paper was discussed by Drs. Thornbury, Smith, Pryor, Kuhlman, Grosvenor, Tobie, Jones and Bennett.

The section adjourned at 11 o'clock P. M.

